# National characteristics associated with prevalence of depression and anxiety symptoms: a cross-sectional ecological study

**DOI:** 10.1017/gmh.2022.9

**Published:** 2022-02-18

**Authors:** Anthony F. Jorm, Roger T. Mulder

**Affiliations:** 1 Melbourne School of Population and Global Health, University of Melbourne, Parkville, Victoria 3010, Australia; 2 Department of Psychological Medicine, University of Otago, Christchurch, New Zealand

**Keywords:** Anxiety, cross-national, depression, human development, prevalence

## Abstract

**Background:**

Cross-national comparisons of the prevalence of mental disorders have relied on lay-administered interviews scored using complex diagnostic algorithms. However, this approach has led to some paradoxical findings, with more vulnerable countries showing lower prevalence, and its appropriateness for cross-national comparisons has been questioned. This study used an alternative method involving simple questions from social surveys to assess the prevalence of specific depression and anxiety symptoms, and investigated their association with national indicators of human development, quality of government, mental health resources, and mental health governance.

**Methods:**

The study used data on the prevalence of three symptoms indicating depression or anxiety: sadness, worry, and unhappiness. These data were taken from the Gallup World Poll (142 countries) and the World Values Survey (77 countries). National characteristics examined covered indicators of human development (income, life span, education, gender equality), quality of government (human freedom, perceptions of corruption), mental health resources (per capita numbers of psychiatrists, mental health nurses, psychologists, and social workers), and mental health governance (whether there is a national mental health plan and a mental health law).

**Results:**

All the human development and quality of government indicators, and some of the mental health resource indicators, were strongly associated with a lower prevalence of symptoms.

**Conclusion:**

Populations of nations with higher human development, quality of government, and mental health resources have better mental health when measured by the prevalence of specific symptoms.

## Introduction

Cross-national comparisons of the prevalence of mental disorders have the potential to increase understanding of the factors that determine a nation's mental health. The ability to carry out these studies received a considerable boost with the development of standardized psychiatric interviews in the 1980s that could be administered by lay interviewers and scored with diagnostic algorithms. The Composite International Diagnostic Interview (CIDI), in particular, was specifically designed for cross-national studies and has underpinned much of the research in the area. However, concerns have been expressed about the validity of cross-national comparisons of prevalence using the CIDI. These concerns have been based on the possibility of measurement error due to national differences in stigma and somatization (Patten, 2003), cultural differences in the degree to which symptoms impair functioning (e.g. in some cultures, people may be more prone to ‘soldier on’) (Mulder *et al*., 2020), and a greater willingness by people from Western countries to endorse screening questions even when they do not have as severe symptoms (Scorza *et al*., 2018).

There has also been skepticism about the findings from these studies, given that low-income countries and those affected by greater trauma exposure have been found to have equal or lower prevalence of common mental disorders compared to other countries (Patten, 2003; Jorm, 2006; Vermetten *et al*., 2016). In particular, lower prevalence has been found in countries with a higher Vulnerability Index, which is a complex aggregation of 23 indicators, covering Susceptibility (public infrastructure, nutrition, poverty and dependences, economic capacity and income distribution), Coping (government and authorities, medical services, material insurance coverage) and Adaptation (education and research, gender equality, environmental status/ecosystem protection, health investment). Such observations have led to the concept of a ‘vulnerability paradox’, where greater vulnerability is associated with a higher risk of disorders for individuals within a country, but a lower risk at a national level between countries (Dückers *et al*., 2016, 2019; Dückers and Olff, 2017). This paradox has also been questioned as to its plausibility (Vermetten *et al*., 2016; Mulder *et al*., 2020).

We have proposed an alternative simpler method of making cross-national comparisons of mental health, which avoids the need for complex branching interviews involving questions about the timing, duration, and impact of symptoms and the use of diagnostic algorithms (Jorm, 2006; Mulder *et al*., 2020). This method involves examining the prevalence of individual symptoms assessed in social surveys of representative samples. We have recently used this method to investigate cross-national differences in the prevalence of sadness, worry, and unhappiness (Jorm and Mulder, 2021). The justification for using these symptoms is that they are frequently included in scales measuring depression, anxiety, or general mental health (see online Supplementary File 1, which lists nine scales which ask about sadness, 11 which ask about worry, and 12 which ask about unhappiness). Using this method, we found a higher prevalence of each of these symptoms in countries with greater vulnerability (as measured by the Vulnerability Index), lower Gross Domestic Product (GDP) per capita, and greater income inequality (as indicated by the Gini Index, which measures inequality on a 0–1 scale). These cross-national associations are consistent with those at the individual level and do not support the existence of a vulnerability paradox.

In the present paper, we aim to use this methodology to investigate other factors that might be associated with national mental health. The characteristics we examined covered human development, quality of government, mental health resources, and mental health governance indicators.

The human development indicators examined covered income, lifespan, education, and gender equality. These were chosen because within countries, the prevalence of mental disorders and symptoms is higher in people who are poor (Ridley *et al*., 2020), less educated (Chlapecka *et al*., 2020), physically unwell (Moussavi *et al*., 2007), and experiencing gender inequality (Van de Velde *et al*., 2013). We hypothesized that these factors would also be associated with a higher prevalence at the country level.

The quality of government indicators examined covered freedom and corruption. Freedom was chosen because a previous study on seven European countries and the USA found that a freedom index was associated with lower symptoms of depression, anxiety, and stress, which the authors attributed to greater autonomy and opportunities to pursue happiness (Scholten *et al*., 2017). Previous research on European countries has also found an association of perceived corruption with depressive symptoms, but that association was non-significant once national wealth was controlled (van Deurzen, 2017). Van Deurzen (2017) has argued that corruption could affect mental health in various ways, including acting as a stressor, decreasing optimism and locus of control, and increasing powerlessness. We hypothesized that countries with greater freedom and less corruption would have a lower prevalence of symptoms.

The mental health resource indicators examined involved the per capita availability of mental health professionals, while the quality of mental health governance was assessed by whether a country had a stand-alone policy or plan for mental health and whether there was a stand-alone law for mental health. These resource and governance indicators were selected because they are regarded as key indicators of mental health system development by the World Health Organization (WHO) (World Health Organization, 2021). We hypothesized that countries with greater resources and quality of governance would have a lower prevalence of symptoms.

## Methods

### Data on prevalence of symptoms

Data on the prevalence of sadness and worry were taken from the 2019 Gallup Global Emotions Report (Gallup, 2019), which surveyed representative samples of adults aged 15 and older from 143 countries in 2018, with samples of around 1000 for most countries. The list of countries covered can be found in the datafile given in online Supplementary File 2. Telephone interviews were carried out in countries where telephone coverage represents at least 80% of the population or where this is the customary survey method, and face-to-face interviews were used in other countries. This survey asked questions about 10 positive emotions (in 143 countries) and 10 negative emotions (from 142 countries) experienced in the day before the survey. The two questions analyzed here as indicators of mental ill health are: ‘Did you experience the following feelings during a lot of the day yesterday? How about worry’ and ‘Did you experience the following feelings during a lot of the day yesterday? How about sadness?’. The participants responded ‘yes’ or ‘no’ to these questions. For the Gallup surveys, the translation process started with an English, French, or Spanish version, depending on the region. A translator who was proficient in the original and target languages translated the survey into the target language and then a second translator reviewed the translation against the original version and recommended any improvements.

Data on the prevalence of unhappiness were taken from Wave 7 of the World Values Survey, which covered surveys carried out in 2017–2020. Wave 7 is planned to involve 80 countries, but at the time the data were accessed, it was available in the World Values Survey online database for 77 countries (World Values Survey, 2020). The list of countries covered can be found in the datafile provided (online Supplementary File 2). To be included in the World Values Survey, a country's data had to involve a face-to-face survey of a representative sample of at least 1000 covering all residents of a country aged 18 years and over. The question on happiness (English version) was: ‘Taking all things together, would you say you are; 1. Very happy 2. Rather happy 3. Not very happy 4. Not at all happy’. The prevalence used here was the proportion of the sample responding ‘Not at all happy’. The World Values Survey questions were provided in major languages (English, Arabic, Spanish and Russian) by the World Values Survey Association. National survey teams were responsible for translations into other languages. In any country, the questionnaire was required to be translated into all languages that were the first language for at least 15% of the population.

### Data on human development indicators

The human development indicators were taken from the three dimensions used by the 2019 Human Development Report to construct the Human Development Index (United Nations Development Programme, 2019). These are the Life Expectancy Index, the Education Index, and the Income Index, with each index having a range from 0 to 1, with higher scores reflecting greater human development. These indices were available for 139 of the 142 countries in the Gallup Global Emotions Report and for 74 of the 77 countries in the World Values Survey.

In addition, we used the Human Development Report's Gender Inequality Index, which is comprised of gender differences in health, empowerment and labor market (United Nations Development Programme, 2019). This index ranges from 0 to 1 with higher scores indicating greater inequality. This additional index was included because women typically have a higher prevalence of depression and anxiety symptoms, and this may arguably be influenced by gender inequality. This index was available for 133 of the 142 countries in the Gallup Global Emotions Report and for 71 of the 77 countries in the World Values Survey.

### Data on quality of government

The quality of government was assessed by the Cato institute's 2019 Human Freedom Index and Transparency International's 2019 Corruption Perceptions Index. The Human Freedom Index is ‘a broad measure that encompasses personal, civil, and economic freedom’ and rates countries on a scale from 0 to 10, with high scores indicating greater freedom (Vàsquez and Porčnik, 2019). The Corruption Perception Index ‘aggregates data from a number of different sources that provide perceptions by business people and country experts of the level of corruption in the public sector’ (Transparency International, 2019). The Human Freedom Index was available for 135 countries and the Corruption Perceptions Index for 140 countries out of the 142 countries in the Gallup Global Emotions Report. Both indices were available for 74 of the 77 countries in the World Values Survey.

### Data on mental health resources and governance

Mental health resource and governance indicators were taken from the World Health Organization's Global Health Observatory (World Health Organization, 2021) with data updated to April 2019. The resource indicators were number of psychiatrists, nurses, social workers, and psychologists working in the mental health sector per 100 000 population. The mental health governance indicators were whether or not there was a stand-alone policy or plan for mental health and whether or not there was a stand-alone law for mental health. These indicators were taken for the most recent year available for each country up to 2017. For the 142 countries in the Gallup Global Emotions Report, these indicators were available for 113 countries for psychiatrists, 92 for nurses, 71 for social workers, and 92 for psychologists. For the 77 countries in the World Values Survey, they were available for 61 countries for psychiatrists, 53 for nurses, 37 for social workers, and 52 for psychologists.

### Statistical analysis

The data were analyzed using Comprehensive Meta-analysis V3 (Borenstein *et al*., 2013), with country as the unit of analysis. In using a meta-analysis approach, we are treating each country's survey of symptom prevalence as a separate study that can be combined with other country surveys in a meta-analysis, with countries/studies weighted according to the size of the survey sample. Meta-regression can then be used to investigate the association of country/study characteristics with symptom prevalence. Because the unit of analysis is the country/study, it is possible to use country/study characteristics as covariates in the meta-regression that are taken from data sources other than the symptom prevalence surveys themselves. Thus, in the current analysis, the symptom prevalence data are from countries/studies in the Gallup surveys and the World Values Survey, but the country/study characteristics are taken from other datasets such as the Human Development Report.

The prevalence proportion of unhappiness, sadness, or worry and the sample size were used to produce the logit of the prevalence proportion and standard error for each country. The random-effects model was used. Meta-regressions were run using the following as moderator variables: Life Expectancy Index, Education Index, Income Index, Gender Inequality Index, Human Development Index, Corruption Perceptions Index, number of psychiatrists, nurses, social workers, and psychologists working in the mental health sector per 100 000 population, and the presence of a national mental health policy or plan and the presence of a national mental health law. Scatterplots were used to visualize the associations, with each country survey represented by a circle, with the size of the circle proportionate to the weight of the survey. Associations were regarded as statistically significant if the two-sided *p* value was <0.05.

In order to give examples of countries with very high or very low mental health according to all three symptoms, a principal components analysis was carried out on the logit transformed prevalence proportions, with scores on the first principal component used as an overall indicator of mental health. This combined mental health variable was only available for the 73 countries that had data on all three symptoms. IBM SPSS Statistics 27 was used for this analysis (IBM Corp, 2020).

## Results

The symptom prevalence data on individual countries are shown in the datafile provided as online Supplementary File 2. Descriptive statistics (mean, s.d., skew, and kurtosis) on all variables are given in online Supplementary File 3.

The random-effects meta-analysis found that the prevalence of the three symptoms was: unhappiness 1.5% (95% CI 1.2–1.8), sadness 24.6% (23.2–26.2), and worry 39.3% (95% CI 37.5–41.1).

In the principal components analysis, the first principal component accounted for 63% of the variance and had loadings of 0.89 for sadness, 0.87 for worry, and 0.57 for unhappiness. For countries with data on all three symptoms, the principal component score indicated that the following had the worst mental health (ranked by low score): Iran, Iraq, Nicaragua, Italy, Armenia, Bolivia, Portugal, Bangladesh, Peru, and Philippines. The 10 with the best mental health (ranked by high score) were: Taiwan, Kazakhstan, Poland, South Korea, Sweden, New Zealand, Kyrgyzstan, Czechia, Tajikistan, and Japan.

Table 1 shows the results of the meta-regressions predicting the prevalence of sadness from each of the covariates. All the human development and quality of government indicators were significantly associated with prevalence, as were two of the resources indicators (psychiatrists and nurses) and one of the mental health governance indicators (mental health law).

**Table 1:** Association of country characteristics with the prevalence of sadness

Country characteristic	Number of countries	*Z*-value	2-sided *p* value	*R*^2^ analog
Education	139	−8.71	<0.0001	0.34
Income	139	−9.15	<0.0001	0.35
Life expectancy	139	−7.71	<0.0001	0.29
Gender inequality	133	9.06	<0.0001	0.38
Human freedom	135	−6.39	<0.0001	0.18
Corruption perceptions	140	−7.61	<0.0001	0.25
Psychiatrists per 100 000	113	−5.51	<0.0001	0.21
Nurses per 100 000	92	−3.14	0.0017	0.07
Social workers per 100 000	71	−1.15	0.2501	0.01
Psychologists per 100 000	92	−1.94	0.0522	0.04
Mental health policy or plan	134	1.61	0.1076	0.01
Mental health law	135	−3.26	0.0011	0.06

Figures S1–S3 (online Supplementary File 4) show some illustrative scatterplots. These show the strongest associations in each of the categories of human resources, quality of government and mental health resources, namely associations with Gender Inequality, Corruption Perceptions, and number of psychiatrists per 100 000.

Table 2 shows the results of the meta-regressions predicting the prevalence of worry. The same associations were statistically significant as for sadness. However, for all these variables, the magnitude of the associations was smaller than for sadness.

**Table 2:** Association of country characteristics with the prevalence of worry

Country characteristic	Number of countries	*Z*-value	2-sided *p* value	*R*^2^ analog
Education	139	−5.80	<0.0001	0.17
Income	139	−5.00	<0.0001	0.13
Life expectancy	139	−3.57	0.0004	0.07
Gender inequality	133	5.29	<0.0001	0.16
Human freedom	135	−4.16	<0.0001	0.07
Corruption perceptions	140	−4.30	<0.0001	0.09
Psychiatrists per 100 000	113	−3.69	0.0002	0.09
Nurses per 100 000	92	−3.01	0.0026	0.06
Social workers per 100 000	71	0.24	0.8115	0.00
Psychologists per 100 000	92	−0.36	0.7223	0.00
Mental health policy or plan	134	1.74	0.0820	0.02
Mental health law	135	−3.06	0.0022	0.06

Table 3 shows the results of the meta-regressions predicting the prevalence of unhappiness. Again, all of the human development and quality of government indicators were significantly associated with prevalence. However, the only resources indicator with a significant association was number of psychiatrists, while neither of the mental health governance indicators was associated. As with sadness, the magnitude of the associations was generally higher than for worry.

**Table 3:** Association of country characteristics with the prevalence of unhappiness

Country characteristic	Number of countries	*Z*-value	2-sided *p* value	*R*^2^ analog
Education	74	−2.91	0.0037	0.14
Income	74	−2.50	0.0126	0.19
Life expectancy	74	−3.62	0.0003	0.24
Gender inequality	71	3.00	0.0027	0.23
Human freedom	74	−3.60	0.0003	0.26
Corruption perceptions	74	−3.49	0.0005	0.31
Psychiatrists per 100 000	61	−2.85	0.0044	0.22
Nurses per 100 000	53	−1.44	0.1489	0.09
Social workers per 100 000	37	−0.86	0.3888	0.02
Psychologists per 100 000	52	−1.69	0.0913	0.13
Mental health policy or plan	71	−0.05	0.9575	0.00
Mental health law	71	−0.02	0.9811	0.00

To see whether there were specific associations that accounted for most of the cross-country variation, a series of exploratory multiple meta-regression analyses was carried out. However, in general, once the most highly correlated covariate was entered into the regression, the remaining covariates accounted for no additional variance. An important reason was that there were high correlations among the human development, quality of government, and some of the mental health resources indicators, as shown in Table 4, resulting in multicollinearity in meta-regression analyses. To give an example, the three best individual predictors of the prevalence of sadness were Gender Inequality (*R*^2^ analog = 0.38, *p* < 0.0001), Income (*R*^2^ analog = 0.35, *p* < 0.0001), and Education (*R*^2^ analog = 0.34, *p* < 0.0001). When all three of these predictors were entered simultaneously in a meta-regression, the overall prediction was the same as for Gender Inequality alone (*R*^2^ analog = 0.38, *p* < 0.0001). The regression coefficient for Gender Inequality was still statistically significant in the simultaneous analysis, but with a higher *p* value (*p* = 0.0326), whereas the coefficients for Income and Education became non-significant. The reason for these reduced *p* values for the individual coefficients is that the three predictors were highly associated, with correlations ranging from 0.86 to 0.89, indicating that it was not possible to isolate the contribution of any one of these predictors independent of the others.

**Table 4:** Correlations among covariates in the Gallup Survey countries: Pearson correlation (*N*)

Covariate	Education	Income	Life expectancy	Gender inequality	Human freedom	Corruption perceptions	Psychiatrists per 100 000	Nurses per 100 000	Social workers per 100 000	Psychologists per 100 000	Mental health policy or plan	Mental health law
Education	1.00 (139)	0.89 (139)	0.83 (139)	−0.88 (133)	0.70 (134)	0.70 (138)	0.67 (113)	0.54 (92)	0.28 (71)	0.40 (92)	0.07 (134)	0.36 (135)
Income		1.00 (139)	0.85 (139)	−0.86 (133)	0.70 (134)	0.73 (138)	0.63 (113)	0.55 (92)	0.27 (71)	0.38 (92)	0.04 (134)	0.29 (135)
Life expectancy			1.00 (139)	−0.87 (133)	0.65 (134)	0.69 (138)	0.60 (113)	0.53 (92)	0.28 (71)	0.41 (92)	0.10 (134)	0.26 (135)
Gender inequality				1.00 (133)	−0.72 (131)	0.76 (133)	−0.70 (110)	−0.58 (90)	−0.25 (69)	−0.36 (89)	−0.03 (129)	−0.28 (130)
Human freedom					1.00 (135)	0.79 (135)	0.67 (110)	0.51 (89)	0.34 (71)	0.39 (89)	0.15 (130)	0.18 (131)
Corruption perceptions						1.00 (140)	0.75 (113)	0.62 (92)	0.41 (71)	0.50 (92)	0.09 (134)	0.20 (135)
Psychiatrists per 100 000							1.00 (113)	0.70 (90)	0.32 (70)	0.57 (91)	0.02 (111)	0.12 (112)
Nurses per 100 000								1.00 (92)	0.24 (62)	0.39 (77)	0.06 (90)	0.03 (91)
Social workers per 100 000									1.00 (71)	0.56 (63)	0.08 (69)	−0.03 (70)
Psychologists per 100 000										1.00 (92)	0.06 (90)	0.06 (91)
Mental health policy or plan											1.00 (134)	0.15 (134)
Mental health law												1.00 (135)

Because symptom prevalence may be affected by the age structure of a population, meta-regressions were also carried out using the percentage of population aged 15–64 and 65+ years (with 0–14 as the reference category) as covariates. However, these variables were found to be strongly related to the Life Expectancy Index, with a multiple *R* of 0.89, implying that the age structure is essentially redundant after the inclusion of Life Expectancy.

## Discussion

As hypothesized, the prevalence of all three symptoms was associated with the indicators of a country's human development. Specifically, there was better mental health in countries with a high level of income and education, long life expectancy, and high gender equality. These cross-country associations are entirely consistent with findings for individuals within countries. There is no evidence of a ‘vulnerability paradox’ in these symptom data. The strength of these associations is large and highly significant, particularly with sadness, accounting for over a third of the variance between countries.

Also as hypothesized, the prevalence of all three symptoms was related to the indicators of quality of government, being lower in countries with more freedom and less corruption. As with the human development indicators, the associations are large and highly statistically significant. For the symptom of sadness, they accounted for over a third of the between-country variance.

The mental health resources indicators showed a more complex pattern of associations that was not always as hypothesized. Number of psychiatrists was consistently associated with a lower prevalence of all three symptoms, whereas number of nurses was associated only with a lower prevalence of sadness and worry, and number of social workers and psychologists was not associated with any symptoms. However, there were considerable missing country data on these professional resources, which reduced the statistical power to detect associations. The data on psychiatrists may be more reliable than for the other professions, as most countries will have medical registers. It is noteworthy that while the number of psychiatrists per 100 000 was a highly significant individual predictor, it was also highly correlated with the other predictors. For example, once the Income Index was controlled, there was no association with the number of psychiatrists, indicating that wealthier countries have better mental health and spend more on psychiatrists.

The mental health governance indicators had the weakest associations, with the existence of a mental health law being significantly associated with a lower prevalence of sadness and worry, but not with unhappiness. Contrary to hypothesis, the existence of a mental health policy or plan was not associated with the prevalence of any of the symptoms.

The weaker associations between mental health resources and symptom prevalence are consistent with concerns that increasing provision of treatment may not be associated with improved mental health (Jorm *et al*., 2017). Data from a range of high-income countries show that large increases in pharmacological and psychological treatment have not led to detectable reductions in the prevalence of psychological distress or the suicide rate (Mojtabai and Jorm, 2015; Mulder *et al*., 2017; Jorm, 2018; Nishi *et al*., 2018; Jorm and Kitchener, 2021). It is possible that identifying and addressing social determinants such as the ones we have reported may be more productive than simply increasing mental health treatment resources.

Taken together, these indicators may be associated with the national prevalence of symptoms because they involve stressors that increase distress in individuals, factors that give individuals greater control of their lives, or the resources available to treat mental health problems when these develop. The indicators may be strongly inter-related because improvements in any indicator are conditional on improvements in others, so that they cannot develop in isolation from each other. These indicators may reflect a general latent variable of country development that is associated with a lower prevalence of symptoms. We hypothesize that this general latent variable is a key determinant of a nation's mental health, rather than being a vulnerability factor as implied by the vulnerability paradox. In proposing this causal hypothesis, we acknowledge that the data are correlational, but it seems more plausible that this general latent variable is a driver of national mental health symptoms rather than the reverse. In hypothesizing this causal role, we also acknowledge that there may be other factors that influence a nation's mental health including war, social conflict, natural disasters, parenting practices, the availability of substances, and nutrition.

Previous research on the prevalence of depression and anxiety disorders using the CIDI has found a higher prevalence of Generalized Anxiety Disorder in countries that are wealthier (Ruscio *et al*., 2017) and that economic development is not an important correlate of depression prevalence (Rai *et al*., 2013). Such findings have led to the proposal of a ‘vulnerability paradox’, which posits that vulnerability is a risk factor for individuals within countries, but is associated with lower risk across countries (Dückers *et al*., 2016). By contrast, the present findings, which are based on individual symptoms rather than disorders, align the country data with the individual data, showing that the pattern of risk is the same within and between countries. To reconcile these apparently discrepant findings may require simultaneous investigation of cross-national differences at both the disorder and symptom level.

This study has a number of limitations which must be acknowledged. This is an ecological study and the associations found between countries may not apply within countries. Many of the indices examined involve composites of multiple indicators and it is possible that the associations might differ if more specific indicators were examined as predictors. The emotional states examined here may not be expressed equivalently in various languages, with differences in connotation possibly affecting prevalence. The social acceptability of reporting these emotions in surveys may also differ between cultures. Such effects will add ‘noise’ that reduces the magnitude of the associations examined here. We have attempted to reduce the impact of these factors by looking for consistent patterns of association with all three symptoms and across two international social surveys. Another limitation is that the mental health indicators examined cover only a small number of internalizing symptoms and the findings may not be generalizable to other dimensions of mental health, such as externalizing problems and psychotic disorders, or to the general *p*-factor of psychopathology (Caspi *et al*., 2014). A final limitation is the unknown quality of the data on mental health resource and governance, which is based on reports from individual countries and involved considerable missing data.

## Conclusion

Although it has been suggested that mental disorders are ‘disorders of development’ (Sachs, 2012), using the prevalence of individual symptoms as an indicator of a nation's mental health, it appears that poorer mental health is associated with low development. To reduce symptom prevalence globally requires greater investment in human development and better quality of government, as well as adequate resourcing of mental health services.

## Supplementary material

For supplementary material accompanying this paper visit http://dx.doi.org/10.1017/gmh.2022.9. click here to view supplementary material
