# Supplementary material for: National characteristics associated with prevalence of depression and anxiety symptoms: a cross-sectional ecological study
Source: Glob Ment Health (Camb). 2022 Feb 18;9:65–71. doi: 10.1017/gmh.2022.9 (PMC9806962; doi:10.1017/gmh.2022.9)
Supplement: Supplementary file 1 [file S2054425122000097sup.zip › S2054425122000097sup001.docx]

**Table 1 Sadness items in mental health scales**

| **Scale** | **Item** |
| --- | --- |
| Basic Depression Questionnaire (Guillot-Valdés et al., 2019) | I feel sad |
| Center for Epidemiologic Studies Depression Scale (CES-D) (Radloff, 1977) | I felt sad |
| Clinically Useful Depression Outcome Scale (Zimmerman et al., 2008) | I felt sad or depressed |
| Depression Anxiety Stress Scales (DASS)—Depression Scale (Lovibond and Lovibond, 1995) | I felt sad and depressed |
| Edinburgh Postnatal Depression Scale (Cox et al., 1987) | I have felt sad or miserable |
| Pediatric Symptom Checklist—Youth Report (Duke et al., 2005) | Feel sad, unhappy |
| Profile of Mood States (POMS) Depression Scale (Lorr et al., 1982) | Sad |
| PROMIS Emotional Distress—Depression—Short Form (Reeve et al., 2007) | I felt sad |
| Short Depression-Happiness Scale (Joseph et al., 2004) | I felt sad |

**Table 2. Worry items in mental health scales**

| **Scale** | **Item** |
| --- | --- |
| Edinburgh Postnatal Depression Scale (Cox et al., 1987) | I have been anxious or worried for no good reason |
| Generalized Anxiety Disorder 7-item (GAD-7) Scale (Spitzer et al., 2006) | Worrying too much about different things |
| Generalized Anxiety Disorder Severity Scale (Beesdo-Baum et al., 2012) | Felt anxious, worried, or nervous |
| Generalized Anxiety Disorders Questionnaire—IV (GAD-Q-IV) (Rodebaugh et al., 2008) | Do you experience excessive worry? |
| Geriatric Depression Scale (Yesavage et al., 1982) | Do you frequently worry about the future? |
| Hospital Anxiety and Depression Scale—Anxiety ( Zigmond and Snaith, 1983) | Worrying thoughts go through my mind |
| Pediatric Symptom Checklist—Youth Report (Duke et al., 2005) | Worry a lot |
| Penn State Worry Questionnaire (Meyer et al., 1990) | I worry all the time |
| Spence Children’s Anxiety Scale (Spence, 1998) | I worry about things |
| State-Trait Anxiety Inventory (Gaudry et al., 1975) | I am worried |
| Strengths and Difficulties Questionnaire-- Emotional Symptoms Scale (Goodman, 1997) | I worry a lot |

**Table 3. Unhappiness items in mental health scales**

| **Scale** | **Item** |
| --- | --- |
| Basic Depression Questionnaire (Guillot-Valdés et al., 2019) | I feel unhappy |
| Center for Epidemiologic Studies Depression Scale (CES-D) (Radloff, 1977) | I was happy |
| General Health Questionnaire—12 item (GHQ-12) (Goldberg et al., 1997) | Been feeling reasonably happy, all things considered |
| Geriatric Depression Scale (Yesavage et al., 1982) | Do you feel happy most of the time? |
| Mental Health Inventory (Veit and Ware, 1983) | How happy, satisfied, or pleased have you been with your personal life during the past month? |
| Mood and Feelings Questionnaire-Self Report (MFQSelf) (Wood et al., 1995) | I felt miserable or unhappy |
| Pediatric Symptom Checklist—Youth Report (Duke et al., 2005) | Feel sad, unhappy |
| Profile of Mood States (POMS) Depression Scale (Lorr et al., 1982) | Unhappy |
| PROMIS Emotional Distress—Depression—Short Form (Reeve et al., 2007) | I felt unhappy |
| SF-36 Mental Health Sub-Scale (Ware and Sherbourne, 1992) | Have you been happy? |
| Short Depression-Happiness Scale (Joseph et al., 2004) | I felt happy |
| Strengths and Difficulties Questionnaire-- Emotional Symptoms Scale (Goodman, 1997) | I am often unhappy, depressed or tearful |

**References**

**Beesdo-Baum K, Klotsche J, Knappe S, et al.** (2012) Psychometric properties of the dimensional anxiety scales for DSM-V in an unselected sample of German treatment seeking patients. *Depression and Anxiety* **29**, 1014-1024.

**Cox JL, Holden JM and Sagovsky R** (1987) Detection of postnatal depression. Development of the 10-item Edinburgh Postnatal Depression Scale. *British Journal of Psychiatry* **150**, 782-786.

**Duke N, Ireland M and Borowsky IW** (2005) Identifying psychosocial problems among youth: factors associated with youth agreement on a positive parent-completed PSC-17. *Child: Care, Health and Develoment* **31**: 563-573.

**Gaudry E, Vagg P and Spielberger CD** (1975) Validation of the State-Trait Distinction in Anxiety Research. *Multivariate Behavioral Research* **10**, 331-341.

**Goldberg DP, Gater R, Sartorius N, et al.** (1997) The validity of two versions of the GHQ in the WHO study of mental illness in general health care. *Psychological Medicine* **27**: 191-197.

**Goodman R** (1997) The Strengths and Difficulties Questionnaire: a research note. *Journal of Child Psychology and Psychiatry* **38**, 581-586.

**Guillot-Valdés M, Guillén-Riquelme A and Buela-Casal G** (2019) Reliability and validity of the Basic Depression Questionnaire. *International Journal of Clinical and Health Psychology* **19**, 243-250.

**Joseph S, Linley PA, Harwood J, et al.** (2004) Rapid assessment of well-being: The Short Depression-Happiness Scale (SDHS). *Psychology and Psychotherapy* **77**, 463-478.

**Lorr M, McNair DM and Fisher S** (1982) Evidence for Bipolar Mood States. *Journal of Personality Assessment* **46**, 432-436.

**Lovibond PF and Lovibond SH** (1995) The structure of negative emotional states: comparison of the Depression Anxiety Stress Scales (DASS) with the Beck Depression and Anxiety Inventories. *Behaviour Research and Therapy* **33**, 335-343.

**Meyer TJ, Miller ML, Metzger RL, et al.** (1990) Development and validation of the Penn State Worry Questionnaire. *Behaviour Research and Therapy* **28**, 487-495.

**Radloff LS** (1977) The CES-D Scale: A Self-Report Depression Scale for Research in the General Population. *Applied Psychological Measurement* **1**, 385-401.

**Reeve BB, Hays RD, Bjorner JB, et al.** (2007) Psychometric evaluation and calibration of health-related quality of life item banks: plans for the Patient-Reported Outcomes Measurement Information System (PROMIS). *Medical Care* **45**, S22-31.

**Rodebaugh TL, Holaway RM and Heimberg RG** (2008) The factor structure and dimensional scoring of the generalized anxiety disorder questionnaire for DSM-IV. *Assessment* **15**, 343-350.

**Spence SH** (1998) A measure of anxiety symptoms among children. *Behaviour Research and Therapy* **36**, 545-566.

**Spitzer RL, Kroenke K, Williams JB, et al.** (2006) A brief measure for assessing generalized anxiety disorder: the GAD-7. *Archives of Internal Medicine* **166**, 1092-1097.

**Veit CT and Ware JE** (1983) The structure of psychological distress and well-being in general populations. *Journal of Consulting and Clinical Psychology* **51**, 730-742.

**Ware JE, Jr. and Sherbourne CD** (1992) The MOS 36-item short-form health survey (SF-36). I. Conceptual framework and item selection. *Medical Care* **30**, 473-483.

**Wood A, Kroll L, Moore A, et al.** (1995) Properties of the mood and feelings questionnaire in adolescent psychiatric outpatients: a research note. *Journal of Child Psychology and Psychiatry* **36**, 327-334.

**Yesavage JA, Brink TL, Rose TL, et al.** (1982) Development and validation of a geriatric depression screening scale: a preliminary report. *Journal of Psychiatr Research* **17**, 37-49.

**Zigmond AS and Snaith RP** (1983) The hospital anxiety and depression scale. *Acta Psychiatrica Scandinavica* **67**, 361-370.

**Zimmerman M, Chelminski I, McGlinchey JB, et al.** (2008) A clinically useful depression outcome scale. *Comprehensive Psychiatry* 49, 131-140.
