# Supplementary material for: National characteristics associated with prevalence of depression and anxiety symptoms: a cross-sectional ecological study
Source: Glob Ment Health (Camb). 2022 Feb 18;9:65–71. doi: 10.1017/gmh.2022.9 (PMC9806962; doi:10.1017/gmh.2022.9)
Supplement: Supplementary file 1 [file S2054425122000097sup.zip › S2054425122000097sup003.docx]

Descriptive statistics on the country variables

| **Variable** | **N of countries** | **Min** | **Max** | **Mean** | **SD** | **Skewness (SE)** | **Kurtosis (SE)** |
| --- | --- | --- | --- | --- | --- | --- | --- |
| Education index | 142 | .222 | .946 | .668 | .180 | -.436 (.203) | -.723 (.404) |
| Income Index | 142 | .285 | 1.000 | .711 | .178 | -.393 (.203) | -.828 (.404) |
| Life Expectancy Index | 142 | .523 | .995 | .817 | .117 | -.540 (.203) | -.576 (.404) |
| Gender Inequality Index | 135 | .037 | .834 | .336 | .198 | .059 (.209) | -1.146 (.414) |
| Human Freedom Index | 138 | 3.800 | 8.880 | 6.980 | 1.103 | -.287 (.206) | -.352 (.410) |
| Corruption Perceptions Index | 143 | 15.000 | 87.000 | 44.105 | 19.232 | .733 (.203) | -.512 (.403) |
| No. psychiatrists per 100,000 | 114 | .007 | 48.040 | 5.850 | 8.644 | 2.378 (.226) | 7.210 (.449) |
| No. nurses per 100,000 | 93 | .000 | 150.300 | 16.871 | 28.933 | 2.585 (.250) | 6.996 (.495) |
| No, social workers per 100,000 | 72 | .000 | 145.400 | 5.237 | 20.346 | 5.651 (.283) | 34.588 (.559) |
| No. psychologists per 100,000 | 93 | .008 | 222.600 | 14.736 | 36.021 | 3.513 (.250) | 14.005 (.495) |
| Stand-alone law | 137 | 0 | 1 | .635 | .483 | -.567 (.207) | -1.703 (.411) |
| Stand-along policy or plan | 136 | 0 | 1 | .824 | .383 | -1.716 (.208) | .960 (.413) |
| Logit of ‘not at all happy’ prevalence | 79 | -6.907 | -1.658 | -4.255 | .929 | .332 (.271) | .918 (.535) |
| Logit of sadness prevalence | 142 | -2.944 | .160 | -1.122 | .523 | -.169 (.203) | .437 (.404) |
| Logit of worry prevalence | 142 | -1.992 | .532 | -.437 | .494 | -.272 (.203) | -.400 (.404) |
