# Supplementary material for: National characteristics associated with prevalence of depression and anxiety symptoms: a cross-sectional ecological study
Source: Glob Ment Health (Camb). 2022 Feb 18;9:65–71. doi: 10.1017/gmh.2022.9 (PMC9806962; doi:10.1017/gmh.2022.9)
Supplement: Supplementary file 1 [file S2054425122000097sup.zip › S2054425122000097sup004.docx]

**Figure 1. Regression of logit prevalence of sadness on the Gender Inequality Index**

**Note: The size of the circles in this scatterplot appear the same because the sample sizes in each country are similar.**

**Figure 2. Regression of logit prevalence of sadness on the Corruption Perceptions Index**

**Note: The size of the circles in this scatterplot appear the same because the sample sizes in each country are similar.**

**Figure 3. Regression of logit prevalence of sadness on the number of psychiatrists per 100,000**

**Note: The size of the circles in this scatterplot appear the same because the sample sizes in each country are similar.**
